# The Dynamical Emergence of Biology From Physics: Branching Causation via Biomolecules

**DOI:** 10.3389/fphys.2018.01966

**Published:** 2019-01-25

**Authors:** George F. R. Ellis, Jonathan Kopel

**Affiliations:** ^1^Mathematics Department, University of Cape Town, Cape Town, South Africa; ^2^Texas Tech University Health Sciences Center (TTUHSC), Lubbock, TX, United States

**Keywords:** hierarchy of emergence, bio-molecules, top-down causation, branching logic, natural selection, voltage-gated ion channels

## Abstract

Biology differs fundamentally from the physics that underlies it. This paper^1^ proposes that the essential difference is that while physics at its fundamental level is Hamiltonian, in biology, once life has come into existence, causation of a contextual branching nature occurs at every level of the hierarchy of emergence at each time. The key feature allowing this to happen is the way biomolecules such as voltage-gated ion channels can act to enable branching logic to arise from the underlying physics, despite that physics *per se* being of a deterministic nature. Much randomness occurs at the molecular level, which enables higher level functions to select lower level outcomes according to higher level needs. Intelligent causation occurs when organisms engage in deduction, enabling prediction and planning. This is possible because ion channels enable action potentials to propagate in axons. The further key feature is that such branching biological behavior acts down to cause the underlying physical interactions to also exhibit a contextual branching behavior.

## 1. Biology vs. Physics

Biology arises out of the underlying physics, but living systems have an essentially different nature than natural systems because *inter alia* they involve purpose or function (Hartwell et al., 1999), information (Nurse, 2008), organization (Mossio et al., 2016), and variation (Montévil et al., 2016). How do they arise from the underlying physics, which has none of these characteristics? Physics and biology are essentially different, even though physics underlies biology. We will identify the physics-biology difference, once life has come into existence, as being due to the fact that biological causation is based at the cellular level in logical branching shaped by context, enabled in physical terms by the nature of particular proteins. Because this branching is controlled in a top down way by physiological conditions (Noble, 2008, 2012, 2016) this leads to contextual emergence (Atmanspacher and beim Graben, 2009), which is a form of strong emergence, enabling branching behavior to also emerge at the higher levels.

### 1.1. The Nature of Physics

Physics deals with laws expressing the inevitable interactions of matter and fields according to boundary and initial conditions, and their consequences for emergent physical systems such as gases, liquids, crystals, rocks, planets, stars, and galaxies.

Classical physics proceeds in a deterministic fashion, described by Hamiltonian dynamics (section 2.1). The interactions proceed in a remorseless impersonal way as described by these laws, with no hint of function or purpose. They can exhibit branching behavior in phase changes, as discussed in section 2.2 below, but there is again no trace of purpose or choice in that behavior. Quantum physics has a branching behavior, but that again is nothing to do with choice or function: it is to do with irreducible randomness of quantum outcomes (section 2.3).

When applied to large collections of particles, statistical physics emerges from these interactions and describes how ensembles of particles behave (Penrose, 1979; Blundell and Blundell, 2008). This gives constraints on biology (England, 2013; Perunov et al., 2016) which are necessary, but are not sufficient in themselves to explain function or purpose as in section 1.2.

### 1.2. The Nature of Biology

Many characterizations of life have been given. They include,

- All life exhibits function or purpose (Hartwell et al., 1999), as discussed in the next section.
- In order that this can arise, there must be organization (Solms and Friston, 2018) in the form of adaptive modular hierarchical structures (Ellis, 2016).
- As well as bottom up emergence of higher level structures and function in that hierarchy, there must be top-down realization of higher level processes (Noble, 2012, 2016; Ellis, 2016; Flack, 2017), enabling same level causation at each level (Noble, 2012) and closure of constraints (Mossio and Moreno, 2010; Montévil and Mossio, 2015), with processes thereby generating their own constraints with a mutual dependence such that they both depend on and contribute to maintaining each other.
- This is all enabled by information flows (Nurse, 2008) and associated cell signaling (Berridge, 2014).
- Adaptation to context is taking place all the time at all levels of the hierarchy through variation and selection (Ellis, 2016; Solms and Friston, 2018)
- In particular it is through evo-devo processes (Carroll, 2005; Müller, 2007; Gilbert and Epel, 2009) that all levels of physiological systems come into being, once life has begun^2^.
- These processes have a very noisy and contingent nature at the lower levels (Montévil et al., 2016), despite which reliable physiological functioning emerges at higher levels (Rhoades and Pflanzer, 1989; Randall et al., 2002).

As summarized by Hartwell et al. (1999):

“*Although living systems obey the laws of physics and chemistry, the notion of function or purpose differentiates biology from other natural sciences. Organisms exist to reproduce, whereas, outside religious belief, rocks and stars have no purpose. Selection for function has produced the living cell, with a unique set of properties that distinguish it from inanimate systems of interacting molecules. Cells exist far from thermal equilibrium by harvesting energy from their environment. They are composed of thousands of different types of molecule. They contain information for their survival and reproduction, in the form of their DNA*.”

To make this happens involves *inter alia* multiple interactions and non-linearities, the coupling of self-assembly and self-organization processes with chemical/metabolic reactions, existence of cyclic networks, modular/hierarchical substructures, compartmentalization, and cellular individualization.

Finally, what is life? Our view will be (cf. Hartwell et al., 1999) that a living system is a material system that exhibits all the characteristics just listed. From now on we will take that for granted.

### 1.3. The Concept of Function

Functional talk is a contested area in the philosophy of biology (Millikan, 1989; Neander, 1991; Amundson and Lauder, 1994; Godfrey-Smith, 1994). It is discussed in depth by Mossio et al. (2009). One cannot sensibly talk about physiology of living systems without talking about function or purpose (Hartwell et al., 1999): the heart exists in order to circulate blood (Randall et al., 2002, p. 476–510), pacemaking cells exist in order to determine the rhythm of the heart, blood exists in order to transport oxygen, mitochondria in eukaryotes provide energy for cell processes by converting sugars to ATP (Randall et al., 2002, p. 74), and so on Rhoades and Pflanzer (1989). This crucial role of many functions is taken for granted by working biologists, as in the Hartwell et al. quote above. We will have in mind below functions that are indeed crucial in enabling survival (e.g., the pumping of blood by the heart), and not just incidental byproducts (e.g., the sound the heart makes while pumping).

This amounts to a physiological definition however, another tradition exists that relates function to its evolutionary origin. Mossio et al. Mossio et al. (2009) state

“*A first tradition, usually labeled ‘etiological’, has tried to justify and naturalize the teleological dimension of functions by appealing to a scientifically acceptable causal explanation. In the mainstream formulation, etiological approaches appeal to a historical selective causal process, through which the existence of current functional traits is the consequence of the selection exerted on the effects of previous occurrences of the trait. A second tradition, called ‘systemic’ or ‘dispositional’, discards the teleological dimension of functional attributions as a relevant explanandum by interpreting functions as causal means-end relations at work in a system. From this second perspective, functions do not explain the existence of the bearer; they refer to current contributions of functional traits to some capacity of the system to which they belong.”*

In our view it is crucial to define function in terms of physiological concepts (the dispositional view) rather than evolutionary ones (the etiological view), because if one goes the latter route it is not easily possible to discuss the issue of drift raised in Kimura (1983) and discussed in depth in Nei (2005). We return to this in section 5.2.

After discussing the options in depth, Mossio et al. (2009) in effect go this route. They propose an organizational account (OA) of functions, as follows:

“*According to the OA, a trait type T has a function if, and only if, it is submitted to organizational closure C in a differentiated self-maintaining system S. This definition implies the fulfillment of three different conditions. Accordingly, a trait T has a function if and only if:*

C1. T contributes to the maintenance of the organization O of S;

C2. T is produced and maintained under some constraints exerted by O;

*C3. S is organizationally differentiated*.”

If such a trait exists, its function will tend to lead to evolutionary success and hence to selection for this trait, which will explain its existence (up to the issue of drift).

We will adopt this account of functions in what follows. Three further points arise: First, it is crucial that function exists at each level of the hierarchy in interrelated ways, as discussed by Farnsworth et al. (2017). They consider a *function* to describe a process (an action) and a *trait* to be a property of a biological system at one level which may enable a function to be performed in relation to another level. This is consistent with the above. Second, the organizational closure mentioned is conditional on top-down constraint or realization occurring as well as bottom-up emergence in the modular hierarchy (Noble, 2012, 2016; Ellis, 2016)^3^This is again implicit in the above.

Finally, the above does not necessarily imply consciousness or intention. However, intention does indeed come into play in the case of conscious animals, when purposive behavior (Mayr, 2004, p. 57), perhaps including deductive causation (section 6), occurs. Its emergence is based on the reliable functioning of the underlying physiological systems in the brain (Randall et al., 2002; beim Graben, 2016). We discuss this in section 6.

### 1.4. The Key Problem

The issue we address in this paper is thus, how does purpose or function emerge from purposeless physics on developmental and functional timescales? How does deterministic physics lead to logical branching enabling function?

At the macro level, this occurs through plastic neural networks (Kandel et al., 2013) and physiological systems (Rhoades and Pflanzer, 1989). At the micro level, it occurs through epigenetic effects (Pigliucci and Müller, 2000; Gilbert and Epel, 2009) mediated by gene regulatory networks (Gilbert and Epel, 2009) and signal transduction networks (Janes and Yaffe, 2006; Berridge, 2014), and synaptic interactions (Kandel et al., 2013). But at the underlying physical level, dynamics is Hamiltonian and does not allow a branching evolution depending on context. How are these compatible with each other? The theme of this paper is that biomolecules are the key enabling these branching processes to happen. They enable turning molecular processes ON or OFF depending on cell signals, (Berridge, 2014), which is determined by the context in which they exist (Noble, 2008, 2011, 2016). As described by Berridge in the Introduction to *Cell Signaling Biology* Berridge (2014):

“*The basic principle of cell signaling pathways are that stimuli (e.g., hormones, neurotransmitters or growth factors) acting on cell-surface receptors relay information through intracellular signaling pathways that can have a number of components. They usually begin with the activation of transducers that use amplifiers to generate internal messengers that either act locally or can diffuse throughout the cell. These messengers then engage sensors that are coupled to the effectors that are responsible for activating cellular responses. .. cell signaling is a dynamic process consisting of ON mechanisms during which information flows down the pathway, opposed by the OFF mechanisms that switch off the different steps of the signaling pathway”* (See Module 1: Figure cell signaling mechanism.)

This is an example of the kind of contextual branching that takes place in biology (section 3.1) and distinguishes it from physics.

Note that this is not the same as saying that biological processes can be considered as computational processes, because it is not implying there is a computation or program of some kind determining the branching choices that are made^4^. It is saying that the branching processes which take place at the lower levels, controlled by a large number of cell signaling processes discussed in depth in Berridge's magisterial text (Berridge, 2014), can be regarded to a very good approximation as Boolean (digital) choice processes governed in a top-down contextual way according to functional need. Thus the core of his discussion is how signals turn a large variety of processes ON and OFF. This is a digital logic, emerging from the underlying physics, that underlies all the higher level processes discussed above (section 1.2); they could not be contextually branching processes (which they are) unless there was the possibility of such branching processes at the underlying molecular and cellular levels. To be sure in practice they are not precisely digital processes, for example ion channels do not precisely behave as ON/OFF channels but rather have are a sigmoidal approximation to such behavior ^5^. Nevertheless that description gives an excellent encapsulation of what occurs, as Berridge discusses, and is used for example by Davies and Walker (2016) and Walker et al. (2016) in Boolean network models of gene regulation in yeast.

However, there is also a major random element at the molecular level introducing statistical variation in happenings at that level. It is then remarkable that these lower level processes produce reliable physiological outcomes at higher levels, such as regular heartbeats and breathing (Rhoades and Pflanzer, 1989), as well as evolutionary convergence to produce physiological function (Natarajan et al., 2016). The view here will be, in accordance with Noble and Noble (2018) that it is precisely this variation at the lower level that allows higher level processes to determine what occurs at the lower levels in order to adapt them to higher level needs (section 5.4). Thus despite this variation one can usefully analyse gene regulation via the above mentioned Boolean network models (Davies and Walker, 2016; Walker et al., 2016), which rely on the kind of branching logic discussed in this paper. Indeed the key point is that

**The lower level basis of higher level contextual functioning:** *None of the complex higher level biological features mentioned in section 1.2 would be possible if there was not a possibility of contextual branching function at the molecular level, which can often be well described by digital (Boolean) logic, despite the statistical nature of molecular processes*.

How that happens is the concern of this paper.

This paper focuses initially on the voltage gated ion channels that underlie neuronal functioning, although the same applies for example to the active sites of enzyme molecules which are complementary to the shape of the substrate. We first consider the difference between the logic of physics (section 2) and the logic of biology (section 3), then the biomolecules that make this difference possible (section 4), and finally how such molecules have come into being (section 5). The processes of deductive causation are discussed in section (6). The conclusion (section 7) clarifies first the three general kinds of causation that occur in biology, and second how contextual biological dynamics causes branching behavior at the underlying physical level. Overall, this is a view of how physics underlies integrative physiology (where everything occurs in a contextual way Noble, 2012, 2016; Ellis, 2016). We take it for granted that living systems are open non-equilibrium systems (Friston and Stephan, 2007). However, that by itself does not suffice to characterize life: a burning candle satisfies those criteria. More is required (section 1.2).

## 2. Logic of Physics

Basic physics evolution is Hamiltonian (section 2.1), and so does not display any branching behavior. However, two aspects of physical laws do exhibit branching: phase changes (section 2.2) and quantum wave function collapse (section 2.3); but neither of these relate to function as characterized above, enabled by branching dynamics. How then does physics enable such branching to emerge? Through symmetry breaking (section 2.4), which is how quite different behavior can emerge from the underlying physics. In a biological context where higher level branching dynamics occurs, that leads to branching physical behavior at the electron level, as discussed in section 7.2.

### 2.1. Classical Dynamics

Classical physics determines the evolution of a physical system by energy and momentum conservation equations (Arnold, 1989, p. 15–27), a force law (Arnold, 1989, p. 28–50), a Lagrangian (Arnold, 1989, p. 55–61), or a Hamiltonian (Arnold, 1989, p. 65–70,165–266). The context *C* consists of boundary and constraint conditions. The dynamical law uniquely determines later states of the relevant variable **X** from suitable initial conditions **X**(*t*_1_) (Arnold, 1989):

(1) $$\begin{array}{rcl} \text{IF at time }t_{1}\text{,}\text{X} & = & \text{X}\left(t_{1}\right),\text{ THEN at time }t_{2},\\ \;\text{X} & = & H\left(C,\text{X}\left(t_{1}\right),t_{2}\right). \end{array}$$

Here the context *C* is expressed via constraint equations

(2) $$\begin{array}{r} C\left(c,\text{X}\right)=C_{0},dC_{0}/dt=0 \end{array}$$

on the possible values of the variables, with control parameters *c* affecting the form of those constraints. Examples are the dynamics of a classical pendulum (Arnold, 1989), and the gravitational dynamics of celestial objects (Binney and Tremaine, 2008). The dynamic equations have unique solutions, as shown by Arnold (Arnold, 1989, p. 8) (this is a result of *dC*/*dt* = *dC*_0_/*dt* = 0). Thus there is a specific unique outcome: no branching takes place as in (9).

#### 2.1.1. Invariance of Physics

The basic point is that we cannot alter the physical laws that govern what happens. We can however shape outcomes by determining what they act on, for example a pendulum or a digital computer; mathematically this is expressed through the constraints *C*. The physical laws relevant to daily life on Earth are Newton's laws of motion together with Galileo's equations for a falling body and Maxwell's equations for electromagnetism:

(3) $$\begin{array}{rc} \nabla \cdot \text{E}=4\pi \rho , & \nabla \times \text{E}=-\frac{1}{c}\frac{\partial \text{B}}{\partial t}, \end{array}$$

(4) $$\begin{array}{rc} \nabla \cdot \text{B}=0, & \nabla \times \text{B}=\frac{1}{c}\left(4\pi \text{J}+\frac{\partial \text{E}}{\partial t}\right) \end{array}$$

where **E** is the electric field, **B** the magnetic field, ρ the charge, and **J** the current. Nothing can change those interactions. The motion of a particle with charge *e*, mass *m*, and velocity **v** is determined by

(5) $$\begin{array}{r} \text{F}=m\frac{d\text{v}}{dt}=e\left\{\text{E}+\text{v}\times \text{B}\right\}+m\text{g}. \end{array}$$

where **g** is the gravitational field. Equation (1) represents the solutions that necessarily follow from (3–5), proceeding purposelessly on the basis of the context *C*. These equations are time symmetric and imply energy conservation. Bifurcations can occur in some cases when a small change in a contextual parameter or initial data occurs, but the outcomes are still determined uniquely by the dynamical equations (Arnold, 1989), even though the outcomes may be unpredictable in practical terms in the case of chaotic dynamics.

Statistical physics laws for aggregates of particles follow from the fundamental physics laws (Penrose, 1979; Blundell and Blundell, 2008), which emergent laws by their nature determine probabilistic outcomes *P*(*q*) for states *q*. They may also have stochastic elements due to random environmental effects, leading to stochastical dynamics represented by coupling deterministic equations of motion to “noise” that mimics the effect of many unknown variables. Then a stochastic term η(*t*) must be added to (5) (see Longtin, 2010). The outcome will then not be determinate, but it will not relate in any way to function or purpose.

### 2.2. Phase Changes

One might suggest that bifurcations as proposed below (Equation 9) happen in physics when phase changes takes place, for example solid/liquid/gas transitions for a substance *S* (Blundell and Blundell, 2008). These generically have a form like

(6) $$\begin{array}{r} \text{GIVEN pressure }P\text{and temperature T },\\ \text{ IF}\left\{P,T\right\}\in S_{P,V}\text{THEN S is solid},\\ \text{ELSE IF}\left\{P,T\right\}\in L_{P,V}\text{ THEN S is liquid},\\ \text{ ELSE S is gaseous}. \end{array}$$

Here the context is represented by the pressure *P* and temperature *T*, and *S*_*P, V*_, *L*_*P, V*_ and *G*_*P, V*_ are the subsets of the (*P, V*) plane for solids, liquids, and gases respectively. At first glance this looks like it has the biological branching form (9). However, the regions *S*_*P, V*_, *L*_*P, V*_, and *G*_*P, V*_ are fixed by the physics of the substance. Thus this is physical logic, determined purely by the laws of physics; no historical or evolutionary factor enters. Note for example the contrast with the homeostatic process governing core body temperature, where the setpoint of 98.4^*o*^F is not determined by physical laws; it was determined through evolutionary processes related to physiological optimization.

### 2.3. Quantum Physics

The Schrödinger evolution is Hamiltonian, but wave function collapse, as occurs when a measurement takes place, is a branching operation. However, such wave function collapse of a wave function |Ψ(*t*_1_)〉 (an “event”) is not deterministic. It has the logic

(7) $$\begin{array}{l} \;\text{IF}\;|\Psi (t_{1})\rangle =c_{1}|u_{1}\rangle +c_{2}|u_{2}\rangle +....+c_{n}|u_{n}\rangle ,\\ \;\text{THEN}\;|\Psi (t_{2})\rangle =\text{EITHER}\;a_{1}|u_{1}\rangle \;\text{OR}\;a_{2}|u_{2}\rangle ....\;\text{OR}\;a_{N}|u_{N}\rangle \\ \text{with probabilities}\;|c_{1}{|}^{2},|c_{2}{|}^{2},...,|c_{N}{|}^{2}\text{respectively}. \end{array}$$

where *a*_*i*_ is the eigenvalue associated with the basis vector |*u*_*i*_〉. Thus branching takes place, but the outcome that occurs is not fixed by the initial state, although the statistics of such outcomes is. It is a contextual process (Drossel and Ellis, 2018), but the logic (7) is not directly related to function. In the end all the processes we discuss in this paper are underlain by such contextual quantum-to-classical transitions.

### 2.4. Symmetry Breaking

The key physical effect enabling the existence of the biomolecules discussed here, with their functional properties arising out of complex molecular structures, is the existence of *broken symmetries* (Longo et al., 2012). These are what allow quite different kinds of behavior to emerge at higher levels out of the underlying physical laws, with all their symmetry properties, as explained by Anderson in his foundational paper “More is Different" (Anderson, 1972). Thus the underlying standard model of particle physics is Lorentz invariant, but the emergent biomolecules (such as shown in **Figures 3**, **4**) are not. Contextless physics is Hamiltonian, but physics in a biomolecular context is not (section 7.2). Hence in the end this is what enables the difference between physics and biology.

Again the underlying physics relevant to biological functioning is time symmetric, but biological effects such as cell signaling (Berridge, 2014) and adaptive selection (18) are not. The contextual process of wave function collapse in quantum physics (7) breaks the time symmetric of the Hamiltonian evolution of the wave function, and this underlies the way the cosmological arrow of time leads to the arrows of time in quantum physics and thermodynamics (Drossel and Ellis, 2018), and so underlies the crucial feature of the emergence of the arrow of time in biology. We will not comment further on this issue here.

## 3. Logic of Life

Life of course obeys the laws of physics, so at each level whatever constraints are implied by physics are obeyed (Cockell, 2018). However, additionally living systems behave according to biological logic, leading to what Mayr characterizes as goal directed behavior (Mayr, 2004, p. 52) furthering function (section 1.3). Living systems collect and analyse information (Nurse, 2008), using it to predict probabilities and thereby use it to execute functional actions in the light of both genetic heritage and acquired information (Hartwell et al., 1999; Campbell and Reece, 2005). This involves a branching logic where outcomes are selected on the basis of context, as revealed by incoming information.

### 3.1. Dynamical Branching

The dynamics followed at each level of biological hierarchies is based on contextually informed dynamical branching *L* that support the functions α of a trait *T*. Thus biological dynamics can be functionally-directed rather than driven by inevitability or chance:

(8) $$\begin{array}{r} \text{Biological\#x000A0;dynamics tends to further}\\ \text{the function}\alpha \text{of a trait}T\\ \text{through contextually informed branching}\\ \text{dynamics}L \end{array}$$

where function is defined as in section 1.3, and in its simplest form *L* is branching logic of the form

(9) $$\begin{array}{r} \text{L: given context}C,\text{IF }T\left(\text{X}\right)\text{THEN}F1\left(\text{Y}\right),\\ \text{ELSE}F2\left(\text{Z}\right). \end{array}$$

Here **X** is a contextual variable which can have many dimensions, and **Y** and **Z** are also variables that may have many dimensions; they may be the same variables or not. *T*(**X**) is the truth value of arbitrary evaluative statements depending on **X**. It can arise from any combination of Boolean logical operations (NOT, AND, OR, NOR, etc.), perhaps combined with mathematical operations, while *F*1(**Y**) and *F*2(**Z**) are outcomes tending to further the function α. Thus they might be the homeostatic response“If blood sugar levels are too high, release insulin,” or the conscious “If the calculated range of the aircraft as presently fueled is <500 km, add more fuel” (a default unstated “ELSE” is always to leave the status quo).

Together with (8), the crucial point is

**Independence of physics**: *The evaluative function T*(**X**) *and the outcome options F*1(**Y**) *and F*2(**Z**) *are not determined by the underlying physical laws, despite being enabled by them*.

Thus these branching processes are not determined by Newton's laws of motion, Maxwell's equations, Newton's or Einstein's theory of gravity, the fundamental theory of particle physics, or statistical physics. Rather they are shaped by evolutionary or developmental processes (Gilbert, 2006; Gilbert and Epel, 2009) to give highly complex outcomes (Rhoades and Pflanzer, 1989; Campbell and Reece, 2005) resulting from plant or animal phsyiology or animal behavior, or can be conceived by human thought so as to result in planned outcomes (Bronowski, 1973; Harford, 2017). In many cases at the molecular level this branching logic is to a very good approximation of a discrete (digital) nature: this is clear for example in Berridge's discussion (Berridge, 2014) of cell signaling systems. There will in practice be noise and time lags in real situations, leading to more complex contextual dynamics. However, a discrete description such as given by Berridge will adequately capture the causal essence of what is going on at a molecular level from a biological viewpoint (if that were not the case, his magisterial book would not make sense).

In more complex cases, there will be multidimensional spaces of options and responses:

(10) $$\begin{array}{r} \text{L: given context}C,\text{IF }B_{N}\left(\text{X}\right)\text{THEN}F_{N}\left(\text{Y}\right) \end{array}$$

where *B*_*N*_ is the *N*th truth function and *F*_*N*_ is the *N*th response function. The key point is the same: there is an evaluation function *B*_*N*_ independent of the underlying physics, and a branching dynamics *F*_*N*_ that is followed depending on that function. In principle one can take a limit where evaluation outcome is continuous but in practice that is unrealistic: there will always be sensitivity limits to detection or response processes, so that in fact responses will be discrete responses to discrete ranges of input variables. In any case we will give a number of key cases below where the biological dynamics is well represented by (9) and it is the higher level dynamics emerging out of combinations of such operations that need description as in (10). In particular (9) is true for the cell signaling networks described by Berridge Berridge (2014), which are at the heart of much molecular biology.

One can suggest that trivially any dynamics of a physical system can be programmed in terms of branching logic equivalent to (10), so (10) is really not different from (1), but as discussed in detail in Binder and Ellis (2016), physical laws are not the same as programs: a physical law is not an algorithm (it is Newton's *Law of Gravity*, not Newton's *Algorithm for Gravity*). Furthermore, there is no Hamiltonian or Lagrangian that leads to (10), and in the physics case there is no function α associated with the dynamics, as in (8). Physics *per se* is not teleonomic and does not show branching behavior related to function (section 2). That is the import of the plethora of existence and uniqueness theorems for fundamental physics (for the gravitational case, see Hawking and Ellis, 1973) whereby initial data determines a unique outcome in a specific spacetime domain (therefore the dynamics does not have a branching nature). Unlike the case of physical laws, where the relevant interactions cannot be changed or chosen because they are given by Nature and are invariable, the branching interactions (10) can fulfill widely varying biological or social or mental functions or purposes and can be selected for those purposes. Once one has this basic logical branching enabled at the molecular level, it is possible for complex emergence to take place where branching dynamics is possible at higher levels, and information can be causally effective (Nurse, 2008; Walker et al., 2017)^6^.

It is of course not intended here to imply that this kind of causation is deterministic: that is why the word “tends” is used in (8); probabilities may be the best description of the branching logic at play. In particular, chance plays a key role in evolutionary theory (Glymour, 2001; Mayr, 2002) and molecular interactions. Nevertheless such causation is often reliable (Rhoades and Pflanzer, 1989; Randall et al., 2002), for example in the case of the developmental programs which underlie developmental biology (Wolpert, 2002; Gilbert, 2006; Berridge, 2014: Module 8), in the case of molecular machines (Hoffmann, 2012), the systems underlying heart function described by Noble (Fink and Noble, 2008), and the metabolic networks and gene regulatory networks described by Wagner (Wagner, 2017). We take that issue up in section 5.4. In the next sections, we look at various forms the branching logic (9) can take, always taking (8) for granted. Key cases are homeostasis (11) and adaptive selection (18).

### 3.2. Homeostasis

A crucial form of branching logic in biology is implemented in feedback control circuits that are the foundations of *homeostasis* (Ashby, 1956; Rhoades and Pflanzer, 1989; Randall et al., 2002; Campbell and Reece, 2005, p. 8–10). These are basically of the form (Randall et al., 2002, p. 11, Modell et al., 2015)

(11) $$\begin{array}{r} \text{IF}X<X_{MIN}\left(C\right)\text{ THEN }X_{INC}\left(\text{Y}\right),\text{ ELSE IF }X>X_{MAX}\left(C\right)\\ \text{ THEN }X_{DEC}\left(\text{Z}\right) \end{array}$$

where *X*_*INC*_(**Y**) is some operation that increases the value of the target variable *X* through changing the value of the control variable **Y**, and *X*_*DEC*_(**Z**) is some operation that decreases the value of *X* through changing the value of **Z** (which may or may not be the same as **Y**). The default is to leave the situation as is. Note that this is not a simple ON/OFF effect (Modell et al., 2015): it is a mechanism which will tend to correct the value of *X* over time to lie between *X*_*MIN*_(*C*) and *X*_*MAX*_(*C*), with dynamics described by the equations of feedback control systems (Di Steffano et al., 1967; Sauro, 2017), using Laplace transforms to model the system and signals, in contrast to the physics Equations (3–5). The triggering values *X*_*MIN*_(*C*) and *X*_*MAX*_(*C*) are in general dependent on the context (e.g., if the organism is sleeping as against running).

This is a particular case of (9). Note that this is just one part of the complex interacting processes generating their own constraints, immersed in many dimensional interactions. However, (11) undoubtedly occurs at both macro and micro levels as part of this larger set of interactions. Thus such processes control blood pressure and core body temperature at the macro level, and potassium and sodium levels in axons and glucose concentration in extracellular fluid at the micro level ^7^. Because biological homeostatic systems have been tuned through evolutionary processes, they are less subject to instabilities that afflict feedback control systems in general.

### 3.3. The Physical Hierarchy

**Figure 1:**
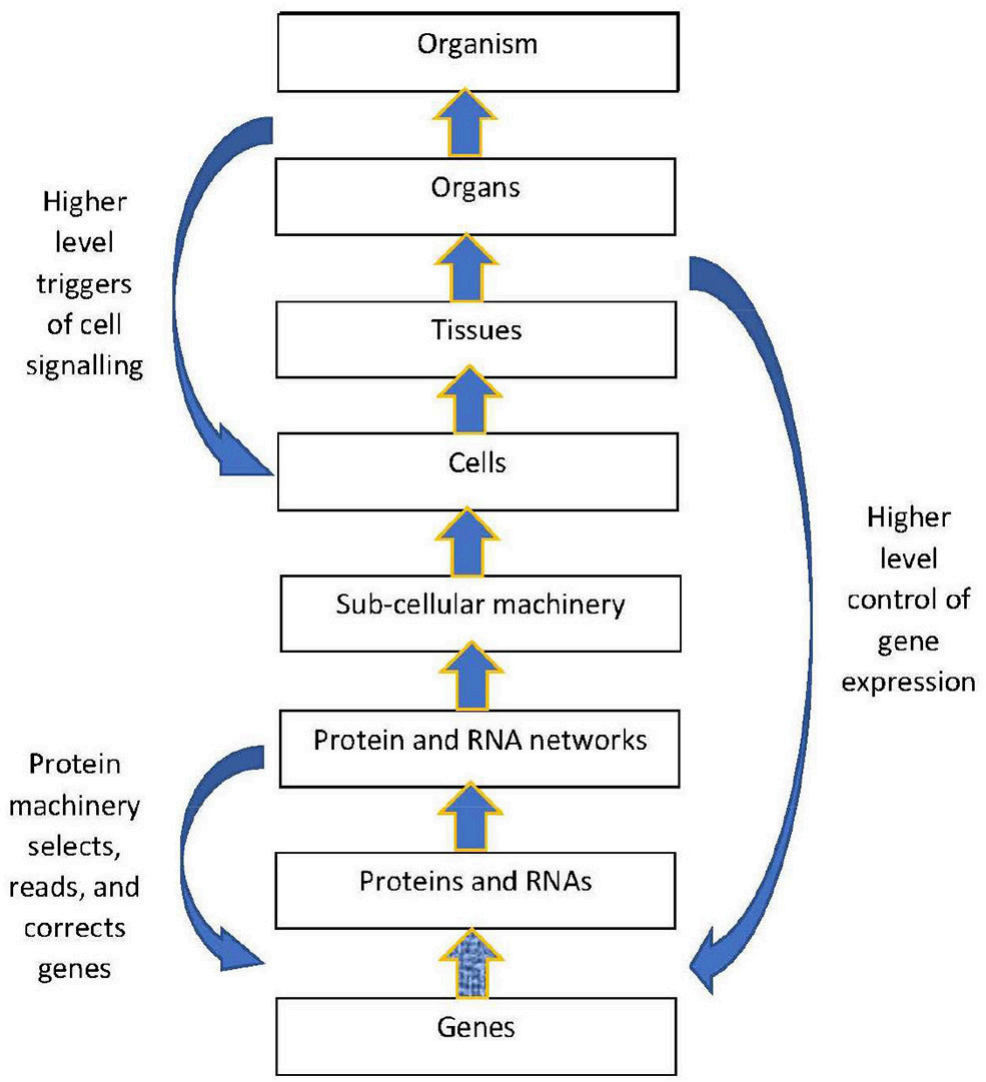
Contextual control in the hierarchy. There is epigenetic control of lower level biological processes by higher level physiological states. These higher states determine what branching will take place at the cellular level by switching genes ON and OFF on the basis of higher level needs. Adapted from Noble (2012), with permission.

The structural hierarchy of life (Ellis, 2016) is indicated in Figure 1. Networks of interactions between lower level modules lead to emergence of higher levels, which in turn act down on the lower levels to shape their interactions (Noble, 2008, 2016; Ellis, 2016). This leads to adaptive same level causation at each level of the hierarchy Noble (2012).

### 3.4. Building the Hierarchy: Black Boxing

Branching dynamics occurs at the molecular and cellular level (Berridge, 2014). When built into cell signaling networks, gene regulatory networks, metabolic networks, and neural networks, this bifurcating dynamics at the lower levels enable emergence of higher order operations such as occur in physiology and the brain, with branching logic (9) or (10) occurring at each level. However, the function of the lower levels is in turn contextually controlled by higher level elements (Noble, 2012), resulting in contextual emergence (Atmanspacher and beim Graben, 2009) where lower level logical choices are set so as to fulfill higher level purpose or function (Noble, 2008, 2012; Ellis, 2016). The combination of bottom-up and top-down effects enables the closure of constraints (Montévil and Mossio, 2015).

Figure 2 from Goelzer et al. (2008) illustrates how branching operations at molecular level in a metabolic pathway can be regulated by higher order circuits through transcription factors that control the transcription of genes. They may be ON (that is, able to bind to DNA) or OFF (Berridge, 2014), in this way controlling transcription of DNA to messenger RNA.

**Figure 2:**
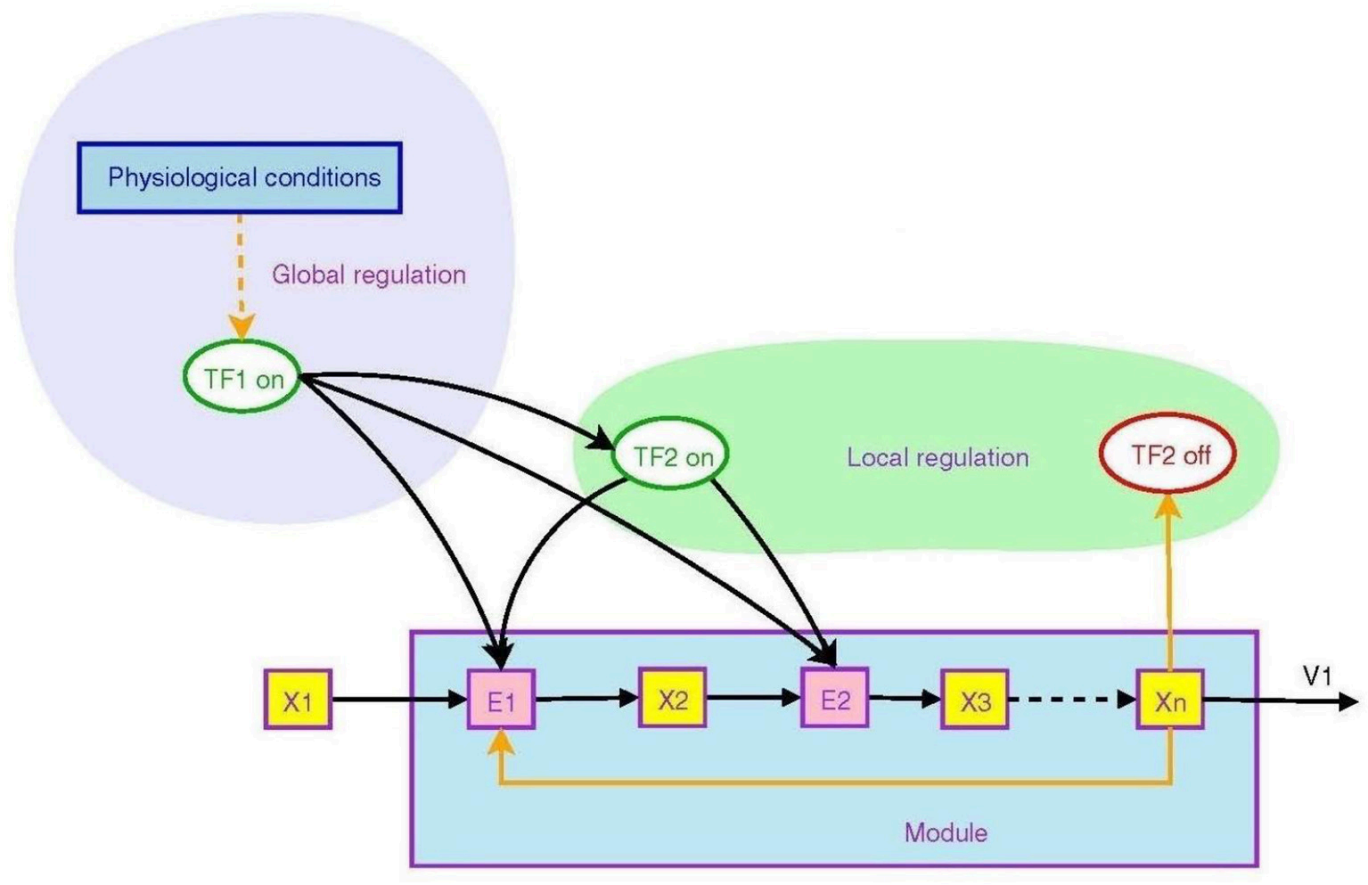
Non-local and local regulation in the context of a metabolic pathway. Depicted is the overall logical structure of a metabolic pathway module that converts food into energy and proteins, with binding of various factors serving as ON/OFF switches. This enables the top-down effect of the physiological environment. Transcription factors are TF's, enzyme pools are E's, metabolic pools are X's. From Goelzer et al. (2008) (open access).

The transcription factor *TF*_2_ is a local variable that is responsive to an intermediate metabolite *X*_*n*_. It modulates synthesis of enzymes in the pathway, embodying branching logic of the form

(12) $$\begin{array}{r} IFTF_{2}on,THENX_{2}\to X_{3},ELSENOT \end{array}$$

which is of the form (9). This is local branching within the module. However, the higher level regulator *TF*_1_, sensitive to variables such as blood pressure or heart rate, modulates the synthesis of both intermediate enzymes and the local transcription factor *TF*_2_. Thus the internal branching of the module results in a “black box” whereby conversion of metabolite *X*_1_ to *X*_*n*_ is determined by the higher level variable *TF*_1_:

(13) $$\begin{array}{r} IFTF_{1}on,THENX_{1}\to X_{n},ELSENOT \end{array}$$

The outcome is again of the branching form (9), but occurring at a higher level (because *TF*_1_ is a higher level variable). The function is production of *X*_*n*_ when and only when it is needed. Thus lower level branching circuits such as (12) can be used to build up higher level branching logic such as (13). This is how *abstraction* occurs in a modular hierarchy (Booch, 1994), so that internal workings of a module are hidden [in this case *TF*_2_, *E*_2_, *X*_2_, and *X*_3_ are internal variables that do not occur in the higher level relation (13)]. From the system view, what matters is the emerging logic (13) where transcription factor *TF*1 controls conversion of metabolite *X*_1_ to *X*_*n*_. Regulation of lower levels through higher level conditions is possible between any adjacent levels in the hierarchy. Through it, metabolic regulation can control gene expression in a top-down way (Alam, 2016), as in Figure 1. The underlying assumption is that there is a suitable cellular context for this to happen (Hofmeyr, 2017).

#### 3.4.1. Black Boxing

As just demonstrated, in the case of a complex logical system, you do not get the higher level behavior by coarse graining, as in the case of determining density and pressure from statistical physics (Penrose, 1979). Instead, you get it by *black boxing* and *logical combination*, involving information hiding and abstraction to characterize the exterior behavior of a module (Ashby, 1960; Oizumi et al., 2014). This is particularly clear in the case of digital computer systems, with their explicit apparatus of abstraction, information hiding, and carefully specified module interfaces, see Grady Booch's book *Object Oriented Analysis* (Booch, 1994). Even though biological systems are not running logical programs, they use the same basic principles of modularity and abstraction in cell signaling systems.

### 3.5. Multiple Realization

A key feature in the emergence of higher level structure and functions is the multiple realization of higher level structures and functions at lower levels. This is central to the way modularity and black boxing works: the function of a module can be realized by many different internal variables and causal networks. Thus in Figure 2, it does not matter what the internal dynamics of the module is provided it leads to the emergent result (13). This degeneracy occurs in all biology in relation to the underlying microbiology and physics: many different lower level realizations of the needed higher level functions can occur. Such multiple realization occurs *inter alia* in the metabolic networks in a cell, gene regulatory networks, and neural networks.

The key underlying analytic concept is existence of *functional* equivalence classes of lower level structures and functions (Auletta et al., 2008; Ellis, 2016) corresponding to a specific emergent structure or function. Equivalence classes at a lower level collect elements whose differences are irrelevant for the emergent target feature at the higher level; it does not matter which one is used to realize the higher level feature. Existence of such functional equivalence classes is an indication of top-down causation (Auletta et al., 2008). An important example is the relation of developmental systems to the genome: a huge number of different genotypes (a *genotype network*) can result in the same phenotype (Wagner, 2017). Any one of these genotypes can be selected for through evolutionary processes in order to lead to a particular emergent function that promotes survival. As far as the higher level function is concerned, it is irrelevant which specific genotype is selected, so it is membership of the equivalence class at the lower level that is the key to what genotype gets selected when adaptation takes place. The huge size of these equivalence classes is what enables adaptive selection to find the needed biomolecules and interaction networks on geological timescales (Wagner, 2017).

## 4. Linking Physics and Biology: The Physical Basis

All these branching operations emerge from the underlying physics, but are of a quite different nature than the deterministic function of physical laws *per se* (section 2). So how is it possible that they can be realized through the functioning of the underlying physical levels? We will now focus on the brain to give the discussion a specific biological context.

### 4.1. The Nervous System

The operations of brains is based in the functioning of neurons that are linked together by synapses, thereby being structured as neural networks (Kandel et al., 2013) enabling neoronal signaling (Berridge, 2014): (Module 10). Spike trains proceed via dendrites to the neuron soma where a summation operation is performed. Spike trains then proceed from the cell body down axons to synapses, where another summation process occurs; signals are passed on to other neurons if the sum is above an activation threshold (Kandel et al., 2013). The function is to underlie the processes of the nervous system that enable an animal to anticipate and counter threats to its existence, thus enhancing its chances of survival.

The flow of currents in the dendrites and axons is determined by the underlying physics, described by equations (3–5) plus statistical relations and diffusion equations. In a neuronal context, these lead to the Hodgkin-Huxley equations (Hodgkin and Huxley, 1952) which characterize how ion flows underlie the existence of action potential spike trains (Randall et al., 2002, p. 132–1139). These equations result from the physical structure of ion channels (Catterall, 2000; Randall et al., 2002, p. 141–150) which control flow of ions in and out of the cell membranes. The constants occurring in these equations are not universal physical constants, but rather are constants that characterize the membrane structure. It is not possible to deduce them from the laws of physics *per se* (Scott, 1995).

### 4.2. Linking Physics to Logic: The Molecular Basis

**Figure 3:**
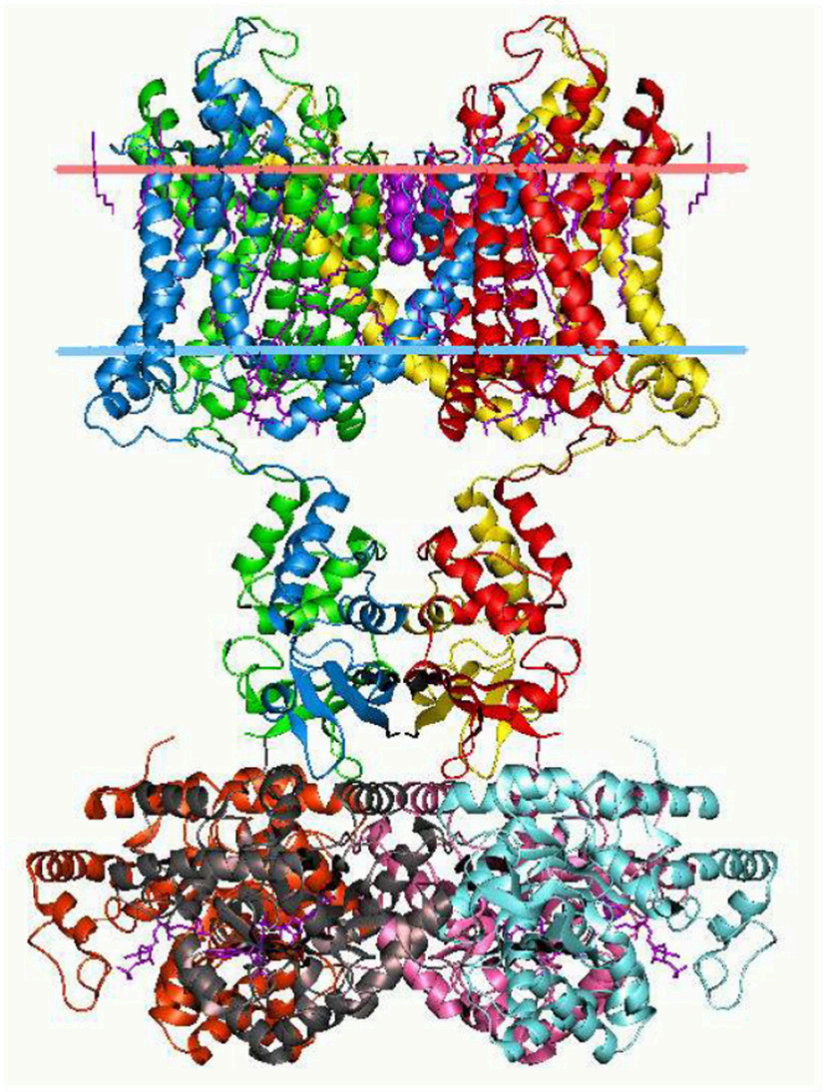
Potassium ion channel structure in a membrane-like environment. This 3-dimensional structure alters according to the voltage difference across the membrane, hence allowing or impeding ion passage. Diagram by Andrei Lomize. From the Open Membranes (OPM) database, with permission.

**Figure 4:**
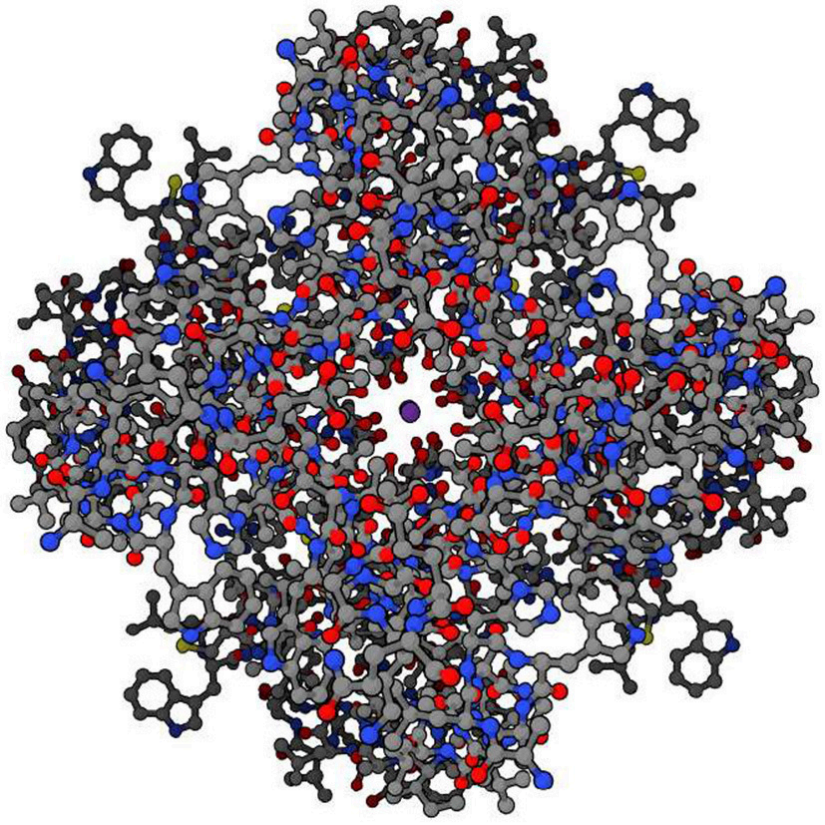
Potassium ion channel functioning. Top view of potassium ion (purple, at center) moving through potassium channel when channel is open. (Protein Data Bank:1BL8, open access).

The branching logical function (10) that emerges is enabled by particular proteins: namely voltage gated ion channels imbedded in axon and dendrite membranes (Catterall, 2000; Randall et al., 2002; Magleby, 2017, p. 146–151) (see Figures 3, 4). They control the flow of potassium, sodium, and chloride ions, leading to action potential spike chain propagation along the axons and dendrites. Their molecular structure and function is discussed in (Randall et al., 2002, p. 139–147).

The ion channels result in branching dynamics with the following logical structure:^8^

(14) $$\begin{array}{r} \text{IF voltage difference }V>V_{0}\text{THEN allow }\\ \text{ion flow , ELSE not} \end{array}$$

which is a specific case of the form (9). The function is to facilitate the propagation of action potentials in axons, and so enable functioning of the nervous system (Randall et al., 2002). It is the detailed 3-dimensional structural form of the ion channels, specifically its tertiary and quaternary structure (see Figures 3, 4), that enables conformational changes in response to local conditions that controls the flow of ions in and out of the cell. This is what enables branching dynamics to emerge from the underlying physics (Farnsworth et al., 2017, p. 313; Kandel et al., 2013; beim Graben, 2016). Similar issues arise via synapses (Kandel et al., 2013; Berridge, 2014: Module 10, p. 28–41), where a branching logic.

(15) $$\begin{array}{r} \text{IF summed input voltage }V>V_{0}\text{THEN fire }\\ \text{action potential , ELSE not} \end{array}$$

holds, enabled by voltage-gated *Ca*^++^ channels in conjunction with pre- and post-synaptic neurotransmitter transporters and post-synaptic receptors.

Once physical implementation of logical processes have been achieved at the lower levels, this provides the building blocks for implementing logical processes at higher levels, enabling emergence of branching function in cortical networks. ON/OFF logical units can be used to give the basic operations AND, OR, NOT, and can then be combined in neural networks with thousands of synaptic connections per neuron, and with both upward and downward connections. This enables the coordinated neural dynamics involved in higher level cognitive functioning. Thus the relevant low level physical structure enabling lower level branching function that then enables emergence of higher level branching function is that of proteins (Petsko and Ringe, 2009) imbedded in the cell membrane.

In summary, Given the right cellular context (Hofmeyr, 2017), biomolecules such as ion channels (Catterall, 2000; Magleby, 2017) can act as logic gates, underlying the emergence of complex life processes where branching logic occurs at the higher levels of physiological systems (Rhoades and Pflanzer, 1989; Campbell and Reece, 2005; Goelzer et al., 2008; Kandel et al., 2013).

### 4.3. More General Biological Contexts

The basic branching logic discussed here occurs also in the metabolic processes, cell signaling networks, and gene expression (controlled by gene regulatory networks) which underlie the functioning of all cells (Berridge, 2014; Hofmeyr, 2017, 2018; Wagner, 2017).

#### 4.3.1. Metabolism

The purpose of metabolism (Krebs, 1993; Berridge, 2014: Module 7; Hofmeyr, 2017) is to produce molecules and free energy needed by the cell in usable form, which are crucial for its function and survival. Enzymes and ribosomes catalyse metaboliism, providing the building blocks of life. This is only possible because of the presence of extremely efficient catalysts, particularly enzymes, that are highly specific with respect to the substrates they recognize and so the reactions they catalyze. The branching logic is (cf. section 3.4),

(16) $$\begin{array}{r} \text{IF catalyst for reaction R1 present }\\ \text{THEN R1 proceeds, ELSE not }. \end{array}$$

Its molecular basis is the relevant lock and key recognition mechanism (Lehn, 1995, 2007; Alberts et al., 2007).

#### 4.3.2. Cell Signaling Networks

These are discussed in depth in Berridge (2014). They are again based in the lock and key recognition mechanism, which at a functional level can be well-described in terms of digital logic as an ON/OFF mechanism (Berridge, 2014). At the molecular level, it is based in complementary molecular shapes (Alberts et al., 2007; Watson, 2013).

#### 4.3.3. Gene Expression and Gene Regulatory Networks

The purpose of the genetic code is to specify the sequence of amino acids that will lead to existence of proteins with crucial cellular functions (Alberts et al., 2007; Watson, 2013). Given the cellular context C (without which no reading of the genetic code would take place Hofmeyr, 2017), the branching logic is

(17) $$\begin{array}{r} \text{IF triplet GGU THEN Gly ELSEIF triplet GGC}\\ \text{THEN Gly ELSEIF …}, \end{array}$$

with a unique mapping specified for each of the 64 codon triplets. Again it is based in complementary molecular shapes that lead to molecular recognition (Watson, 2013). This particular highly degenerate mapping (Watson, 2013; Wagner, 2017) implemented by cellular processes (Alberts et al., 2007) has been determined by the specific historical events of the evolutionary history of life on Earth (Campbell and Reece, 2005; Godfrey-Smith, 2017): many other mappings are chemically possible. Physics by itself does not determine the specific mapping that in fact has occurred (Watson, 2013), represented by the logic (17).

Which sections of DNA are read where and when is under epigenetic control (Carroll, 2005; Gilbert and Epel, 2009), enabled by cell signaling networks (Berridge, 2014) and gene regulatory networks (Wagner, 2017). A key feature of DNA expression is alternative splicing, whereby a single gene codes for multiple proteins, and overlapping genes, where an expressable nucleotide sequence for one gene is also an expressable nucleotide sequence for another. Given epigenetic control that determines these aspects, readout from nucleotide sequences to amino acids takes place as in (17). Furthermore, the epigenetic systems are themselves made up of interacting molecules that arise through the kind of branching logic we discuss here through gene regulatory networks that can be described in a Boolean way to a good approximation (e.g., Wagner, 2011; Davies and Walker, 2016).

## 5. Existence of the Relevant Proteins

Two issues arise here: the possibility of existence of the biomolecules needed, for example those that comprise ion channels, and how they come into being.

### 5.1. The Possibility of Their Existence

Given the nature of physics as we know it (with particular values for the fundamental constants of nature such as the fine structure constant Uzan, 2003), the nature of possible physical structures at the molecular level is controlled by electromagnetism together with quantum physics. Thus the possibility of the existence of biomolecules, and specifically the proteins controlling biological activity (Petsko and Ringe, 2009), is a result of covalent bonds, hydrogen bonds, and van der Waals forces (Watson, 2013).

The result is a space of possible proteins (Petsko and Ringe, 2009) of vast dimensions: an unchanging space of all possible molecular structures (Wagner, 2017). However, their possible existence is not by itself enough: there must be viable mechanisms that can bring them into being.

### 5.2. Their Coming Into Being: Development and Evolution

Given this vast possibility space, how have the specific proteins that actually exist come into existence? This question has developmental and evolutionary aspects.

#### 5.2.1. Developmental and Epigenetic Aspects

The relevant proteins come into being through molecular processes transcribing genetic information coded in DNA (Alberts et al., 2007; Watson, 2013) into amino acid chains, which then fold to create biologically active proteins. This reading of the genotype occurs in a contextual way (Wolpert, 2002; Gilbert, 2006; Noble, 2012) because epigenetic processes (Pigliucci and Müller, 2000; Gilbert and Epel, 2009), controlled by gene regulatory networks, determine which gene segment gets read at a specific time and place, thereby shaping developmental processes according to the local environment (Oyama et al., 2001; Gilbert and Epel, 2009). Epigenetic effects even allow genetic rewriting (Lee et al., 2018) so that “genes are more followers than promoters of evolution” (West-Eberhard, 2003). As stated by Noble and Noble (2017),

“*Organisms and their interacting populations have evolved mechanisms by which they can harness blind stochasticity and so generate rapid functional responses to environmental challenges. They can achieve this by re-organizing their genomes and/or their regulatory networks. Epigenetic as well as DNA changes are involved. Evolution may have no foresight, but it is at least partially directed by organisms themselves and by the populations of which they form part*.”

Nevertheless the reading of the DNA still takes place as above (section 4.3), once epigenetic processes have selected which specific DNA segments will be read in what order.

#### 5.2.2. Evolutionary Aspects

The question then is, how did that genetic information get written? As stated before, we do not enter here into the discussion of how life started: we assume here that somehow cells came into existence, allowing metabolism and the existence and reading of genetic information. In that context, how was it that the genotype for the specific proteins that actually occur (Petsko and Ringe, 2009) come to be written, given that there is a vast space of possible proteins that might have existed (Wagner, 2017)? What about the origin of the gene regulatory networks controlling body plan development (Peter and Davidson, 2011)?

The relevant proteins are extraordinary complex biomolecules (Petsko and Ringe, 2009) with specific functions that are essential for survival, where function is as characterized in section 1.3. For example, hemoglobin transports oxygen in our blood stream; chlorophyll enables plants to harvest solar energy, and so on. Thus they will have been strongly subject to selection pressure because of these vital functions, and so arguably cannot have come into being through genetic mutation, drift, or recombination alone (Morris and Lundberg, 2011, p. 21) without selection playing a decisive role (Farnsworth et al., 2017, p. 313). The natural hypothesis is that they were selected through the process of Darwinian adaptive selection (Darwin, 1872; Mayr, 2002; Campbell and Reece, 2005; Morris and Lundberg, 2011) occurring at the organism level, with these selective outcomes chaining down to the genotyope level within a functional cellular context (Hofmeyr, 2017). The genotype-phenotype map has massive degeneracy that would have played a crucial role in enabling new phenotypes and hence associated genotypes to have come into being in the available time (Wagner, 2011), and doing so in such a way that the organism remains viable at each step. The process is contextually driven and hence is an example of top-down causation (Campbell, 1974; Ellis, 2016).

However caution is warranted. Genetic drift, leading to neutral selection (Kimura, 1983; Lynch and Hill, 1986; Nei, 2005) can explain some aspects of human physiology (Ackermann and Cheverud, 2004; Schroeder and Ackermann, 2017). How do we prove it was selection rather than drift that lead to existence of specific proteins? In the case of phenotypes, one can sometimes determine which features are due to selection pressure and which due to drift, thus a detailed study shows “*during the early evolution of the genus Homo, […] genetic drift was probably the primary force responsible for facial diversification”* (Ackermann and Cheverud, 2004).^9^.

How to determine this for proteins or gene regulatory networks is a fascinating challenge. They obviously play a key physiological role but, particularly given the existence of vast equivalence classes of genotypes that can produce acceptable phenotypes (Wagner, 2011), it is far from clear how to determine what aspects of the proteins are selectively determined and what are due to drift. We simply comment, in agreement with (Wagner, 2017) that there has to have been a major selective aspect underlying their evolutionary development, as otherwise they would not exist able to play the functional roles they do.

### 5.3. The Generic Selection Process

**Figure 5:**
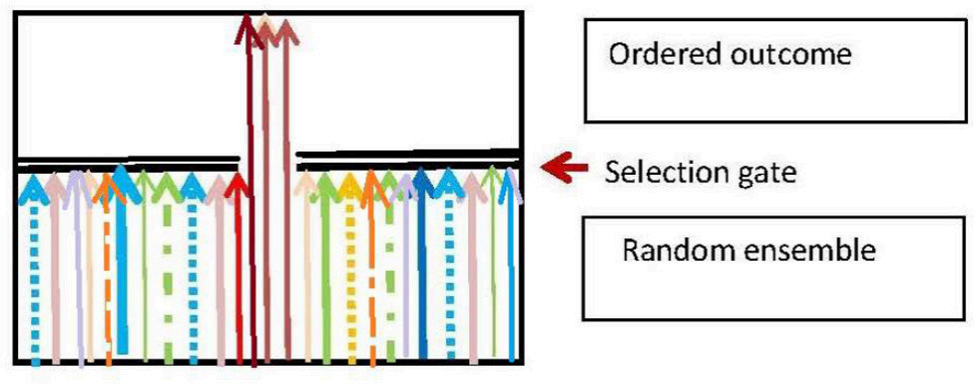
The generic selection process. Adapted from Ellis and Kopel (2018).

Darwinian adaptive selection is a special case of the generic selection process that is ubiquitous in biology. The basic nature of this process is that there is a random input ensemble of entities which is is filtered so as to produce an output ensemble that fulfills some environmentally dependent selection criteria (Figure 5), and so is more ordered than the input ensemble. The branching logic of the process is:

(18) $$\begin{array}{r} \Pi_{S}\left(X\right):\left\{\text{IF}X\notin S\left(C,E\right)\text{THEN delete}X\right\} \end{array}$$

Here *S* is the subset of elements that is selected to survive on the basis of the selection criterion *C*, and the environmental context is E. The resulting effect on the input ensemble {*E*(*X*)} is a projection operation Π_*S*_ that gives the output ensemble {Ê(*X*):

(19) $$\begin{array}{r} \Pi_{S}:\left\{E\left(X\right)\right\}\to \left\{Ê\left(X\right):X\in S\left(C,E\right)\right\}. \end{array}$$

The function of the process is to produce a population of entities that fulfill the selection criterion *C*. The basic physics case is Maxwell's Demon (Von Baeyer, 1998), where the criterion *C* for allowing a molecule to pass the trapdoor is |**v**|>*v*_0_ where |**v**| is molecular speed. A biological case is the immune system, deleting invading pathogens (Rhoades and Pflanzer, 1989; Randall et al., 2002). A logical case is the deletion of emails or files on a computer, in accord with some relevance criterion *C*.

Darwinian selection (Godfrey-Smith, 2001; Mayr, 2002; Campbell and Reece, 2005) has the overall structure (18) where *C* is a measure of inclusive fitness (West and Gardner, 2013) in the context of the environment, and the input ensemble at each time *t*_2_ is a randomized variant of the output of the previous process at time *t*_1_:

(20) $$\begin{array}{r} \left\{E\left(X\right)\right\}\left(t_{2}\right)=R\left\{Ê\left(X\right)\left(t_{1}\right)\right\}. \end{array}$$

At the genotype level, *R* is randomization based in recombination, mutations, and horizontal gene transfer. This results in variation at the phenotype level, which is where the selection (survival of an animal or plant until reproduction can take place) actually occurs^10^; that selection then chains down to the genotype level. Thus the process is a continually repeated multilevel 2-step process (Mayr, 2002: p. 130–133): *reproduction with variation* (20), which is where directed sexual selection and differential reproductive success enters, followed by *elimination* (18), which is where differential survival rates matter (this only requires selection of individuals who are “good enough” (Mayr, 2002, p. 130–131); they don't have to be the fittest, which is partly why drift is possible). It is the elimination phase (18) that leads on average, in suitable circumstances, to selection of individuals with traits that are better fit to the environment. The combination of these two processes leads to inclusive fitness (West and Gardner, 2013). Thus this adaptive selection process (Morris and Lundberg, 2011) functions to produce individuals fit to survive in a specific environmental context through their physiology and functioning even though the process has no intentional “purpose” (Mayr, 2004, p. 58). It thereby leads *inter alia* to existence of the molecules we discuss in this paper (Wagner, 2017).

### 5.4. What Role Does Chance Play?

Biological processes display a great deal of randomness, particularly at the molecular level where there occurs a “molecular storm” (Hoffmann, 2012). The occurrence of this noise does not mean the outcome is random: reliable physiological function emerges at higher levels (Rhoades and Pflanzer, 1989; Randall et al., 2002). In fact microbiology thrives on randomness (Hoffmann, 2012; Noble, 2017), and this is also the case for brain function (Glimcher, 2005; Rolls and Deco, 2010). Furthermore, randomness plays a key role in evolution (see Glymour, 2001; Mayr, 2002, p. 252–254, Kampourakis, 2014, p. 184–191), underlying that vast variety of life on Earth by providing a very varied set of genotypes on which selection can operate, for example leading to predictable convergence in hemoglobin function (Natarajan et al., 2016).

We propose, in agreement with Noble and Noble (2018), that randomness plays a key role at the molecular level by providing an ensemble of variants from which higher level selection processes can choose what happens through selection of outcomes according to higher level selection criteria (18), thus creating order out of disorder in a reliable way (Noble and Noble, 2018), as represented by (19). As stated by Noble and Noble Noble and Noble (2018),

“*Choice in the behavior of organisms involves novelty, which may be unpredictable. Yet in retrospect, we can usually provide a rationale for the choice. A deterministic view of life cannot explain this. The solution to this paradox is that organisms can harness stochasticity through which they can generate many possible solutions to environmental challenges. They must then employ a comparator to find the solution that fits the challenge. What therefore is unpredictable in prospect can become comprehensible in retrospect. Harnessing stochastic and/or chaotic processes is essential to the ability of organisms to have agency and to make choices”*

For example, molecular binding processes depend on random presence of the appropriate substrate for a binding site, and the adaptive immune system depends on random generation of antibodies to find the one that works against a particular pathogen. This is also the essential feature of Edelman's Neural Group Selection (Edelman, 1987), which envisages initial random neuronal connections (Wolpert, 2002) being pruned and strengthened according to selection criteria provided by an innate ‘value system’ in the brain (which in psychological terms can be associated with innate primary emotional systems; Toronchuk and Ellis, 2013; Ellis and Solms, 2017). Furthermore, this underlies the possibiity of real mental emergence, as proposed by Mitchell Mitchell (2018):

“*I argue here that physical indeterminacy provides room for the information entailed in patterns of neuronal firing—the mental content of beliefs, goals, and intentions—to have real causal power in decision-making*.”

## 6. Deductive Causation

Deductive causation takes place when effects are the outcome of explicit logical processes, as contrasted to the biological cases discussed so far, where they are processes that are indeed carrying out what can be characterized as logical operations, but these are implicit in the biology rather than explicit.

Deductive causation requires mental processes that explicitly consider alternative logical inevitabilities or probabilities and decide outcomes on this basis, for example, “If I wait till 10am I will miss the bus, so I'd better leave now”. This requires conscious intelligence^11^, and certainly occurs in the case of humans. It may also occur to some degree in animals, but we will not enter that debate here: the essential point is that it does indeed occur in the real world, as evidenced by the existence of books, aircraft, digital computers, and all the other products of conscious design (Harford, 2017). It is made possible by the existence of brains (at the macro scale) (Kandel et al., 2013) and their underlying biomolecules such as voltage gated ion channels (at the micro scale) (Scott, 1995; Kandel et al., 2013), as discussed in section 4, enabling information to be causally effective (Walker et al., 2017).

We look in section 6.1 at deductive argumentation D, whose truth is valid independent of contingent facts, in section 6.2 at evidence based deduction DE, where the addition of empirical data *E* leads to conclusions that follow from that evidence via logical deduction D, and in section 6.3 at deductively based predictions of outcomes DEO, which are used to decide on best choices of actions DEOC on the basis of logical predictions of outcomes *O* following from the data *E* together with choice criteria C.

### 6.1. Deductive Argumentation

Deductive argumentation can be definite or probabilistic. *Definite deductive arguments* deal with inevitable outcomes of abstract relationships between variables:^12^ thus^13^

(21) $$\begin{array}{r} D:\text{IF }T1\left(\text{X}\right)\text{THEN necessarily }T2\left(\text{Z}\right), \end{array}$$

where *T*1(**X**) may involve logical operations AND, OR, NOT, and their combinations, or mathematical equalities or inequalities, or both logical and mathematical relations in any combination. Thus one might have a conjunction of conditions

(22) $$\begin{array}{r} D2:\text{IF }T1\left(\text{X}\right)\text{AND}T2\left(\text{Y}\right),\text{THEN necessarily }T3\left(\text{Z}\right), \end{array}$$

where **X**, **Y** and **Z** may or may not be the same variables. These are of the same logical form as (9), but the key difference is that in that case, the context was the logic implicitly embodied in biological processes, whereas here the relations refer to explicit logical thought patterns. They may be realized at some moment in a brain, or written down on paper, or recorded in some other way (such as on a black board or a computer screen), but the patterns themselves are abstract relations with their own internal logic that is independent of whatever specific realization may occur.

Mathematical examples are the relations

(23) $$\begin{array}{r} \text{IF \{X=2\} THEN }\left\{\sqrt{X}\text{is irrational }\right\} \end{array}$$

which is proved by algebraic argumentation, and the partial differential equation result

(24) $$\begin{array}{l} \;\text{IF}\;\{\text{Eqns}.(\text{3}),(\text{4})\text{ hold with }\text{J}=\rho =0\},\\ \text{THEN}\;\{\text{wave solutions}\;u(x,t)=F(x-ct)+G(x+ct)\;\text{exist}\} \end{array}$$

(which mathematical fact underlies the existence of radios, TV, cellphones, etc).

Logical examples are the relations

(25) IF{A⇒B}AND{B⇒C}THEN{A⇒C}

and the combinatorial rules of Boolean logic involving AND, OR, NOT, and so on.

*Probabilistic logical arguments* deal with likely outcomes on the basis of statistical evidence, for example:

(26) $$\begin{array}{r} \text{IF }T1\left(\text{X,P1}\right)\text{ AND }T2\left(\text{Y,P2}\right),\text{ THEN probably }T3\left(\text{Z,P3}\right), \end{array}$$

where *T*1(**X, P1**) means *T*1 is valid with probability *P*1, and so on. A key example is Bayes' Theorem (Stone, 2015):

(27) $$\begin{array}{r} \text{IF }\left\{P\left(A\right)\text{AND }P\left(B|A\right)\text{AND }P\left(B\right)\right\}\text{ THEN }P\left(A|B\right)=\frac{P\left(B|A\right)P\left(A\right)}{P\left(B\right)}, \end{array}$$

where *P*(*A*) and *P*(*B*) are the probabilities of observing events *A* and *B* independent of each other, *P*(*A*|*B*) is the conditional probability of observing event *A* given that *B* is true, and *P*(*B*|*A*) is the conditional probability of observing event *B* given that *A* is true. This relation, which is of the form (26), underlies the learning processes of the predictive brain (Huang and Rao, 2011; Clark, 2013; Hohwy, 2013), enabled by suitable neural structures (Hawkins, 2004; Bogacz, 2017, section 2.3–2.5) built from biomolecules (Scott, 1995). This topic is developed further in section 6.5.

### 6.2. The Link to Data: Evidence Based Deduction

It may well be that we know that the antecedents in some of these arguments are either true, or are highly probable, in which case we can move to evidence based deduction: (21) becomes

(28) $$\begin{array}{r} DE:\text{SINCE T1(X) THEN necessarily T2(Z)}, \end{array}$$

where *T*2(*Z*) necessarily follows from *T*1(*X*), and we know *T*1(*X*) to be true either because we have seen it to be true (there is a dog in the room), or it is common knowledge (England is near France), or it is an established scientific fact (DNA is a key molecule underlying genetic inheritance), or at least it is a best explanation (established by abduction, i.e., inference to best explanation from observations). For example

(29) $$\begin{array}{r} \text{SINCE }E=mc^{2}\text{THEN binding energy can be }\\ \text{made available}\\ \text{via nuclear fission of heavy atoms }, \end{array}$$

In other words, because we know special relativity is true, we know we can in principle make nuclear power stations and nuclear bombs. Thus reliable data (the experimental verification of the logically deduced relation *E* = *mc*^2^) relates deductive argumentation to real world possibilities. Similarly an extension of a simple case of (26) becomes

(30) $$\begin{array}{r} \text{SINCE }T1\left(\text{X,P1}\right)\text{THEN probably }T2\left(\text{Z,P3}\right), \end{array}$$

in the probabilistic case, for example

$$\begin{array}{l} \text{SINCE there are dark clouds in the sky }\\ \text{THEN it will probably rain today .} \end{array}$$

The deduction leads to the conclusion that a specific outcome is likely to actually occur.

### 6.3. Deductively Based Action

Following on (28) and (30), we can deductively determine that specific actions will inevitably or probably have specific outcomes:

(31) $$\begin{array}{r} DEO:\text{SINCE }T1\text{is true THEN action A will }\\ \text{ lead to outcome O}. \end{array}$$

This leads to the basis of deductive choice of best actions:

(32) $$\begin{array}{r} DEOC:\text{WHEN }T1\text{is true THEN DO A }(\text{V})\text{ TO}\\ C\text{-optimize O} \end{array}$$

where C is a selection criterion for the best outcome O_*_, and *A*(**V**) is some action chosen to alter *O* via a control variable *V*. The purpose is to produce an optimal outcome *O* on the basis of a representation of the situation founded on the best available evidence (Papineau, 2016). An example is

(33) $$\begin{array}{r} \text{WHEN }\left\{T>T_{0}\right\}\text{ THEN }\{\text{set V ON\} SO THAT }C:\left\{T_{1}<T_{0}\right\} \end{array}$$

which might be part of a computer program implementing feedback control (14) to ensure that temperature *T* is kept below a critical level *T*_0_ via the cooling control variable *V*. In the probabilistic case it might be

$$\begin{array}{l} \text{SINCE \{there is 60\% chance of rain\}}\\ \text{THEN \{take an umbrella\} TO \{keep dry\}.} \end{array}$$

When we carry out such deductive argumentation, the abstract logic of the argument D [see (21)] is the causal element determining the nature of the resulting outcomes. The aircraft flies well because we have used explicit deductive mathematical logic D, together with our knowledge of the laws of fluid dynamics *T*1, to optimize its design O by running computer aided design packages *A*(*V*) representing the aircraft design via variables *V*. We call D a “causal element” because of the counter-factual argument (Menzies, 2014) that if this abstract logic were different, the outcome would be different. The same applies to C: if the decision criteria are changed the outcome changes, for example the wing design will be different if the plane is a fighter or an Airbus. This kind of argument is a key part of planning (Epstude and Roese, 2008).

In practice (e.g., in economic planning) the argument is often probabilistic because we can never be absolutely certain of the outcome, due to uncertainty concerning the contextual effects *C*. Overall, the import of this section is that

**Deductive causation**: *Logical deductions about scientific, engineering, and social issues can lead to action plans that are causally effective in terms of altering the world. In these cases it is explicit abstract logic* D *realized in brains and/or computers that guides and shapes what happens in highly productive ways (Harford*, *2017**) and hence may be said to be the essential cause of what happens*.

This is all possible because of the properties of brains as prediction machines that are also able to make choices between alternatives. The logical operations of deduction D and prediction DEO take place at the psychological level in the brain (Ellis, 2016), while being realized at the neural network level through spike chains, at the axon level through ion flows, and at the electronic level through electron movements (Scott, 1995). Each level does work appropriate to the logic at that level, but it is the high level deductive logic D that determines what happens in terms of specific outcomes through logically based choices DEOC (Ellis, 2016).

### 6.4. The Creative Element

Deductive causation depends on being able to choose between options, which is where imagination comes in. There must be a process in the brain that generates the options that are taken into account when a choice between various options is made:

(34) $$\begin{array}{r} \text{IF }\left\{Thesituation\;is\;S\right\}\text{THEN }\left\{\text{options are}O_{1},O_{2},\ldots ,O_{n}\right\} \end{array}$$

Given this ensemble of choices, one can choose between them using selection criteria as above (section 6.3): a process of adaptive selection takes place whereby an option is chosen, whether it be physical (going to a bus stop, changing a light bulb) or mental (choosing between theories, making a plan). This generation of options to choose from takes place at the psychological level (Byrne, 2005), assisted by the PLAY primary emotional system (Toronchuk and Ellis, 2013; Ellis and Solms, 2017) which is a key source of creativity. There may be an element of randomness in the options available for consideration at the psychological level due to the underlying stochasticity at the neural level (Glimcher, 2005; Rolls and Deco, 2010), in turn due to molecular randomness (section 5.4).

### 6.5. The Adaptive Bayesian Brain

The deductive processes of section 6.1 are determined as valid by the brain through adaptive learning processes leading to logical understanding (Churchland, 2013), enabled by underlying brain plasticity. How does the predictive brain (Hawkins, 2004; Clark, 2013) emerge, whereby the brain estimates prediction errors leading to the Bayesian processes of Equation (27) that then enable learning (Friston, 2018) and prediction (Hohwy, 2013)? This is developed in Friston and Stephan (2007), Buckley et al. (2017), and Bogacz (2017).

which show the mechanism whereby such processes can arise in the brain through neural circuits such as shown in Bogacz (2017). Overall, this all emerges from a network of neurons connected by synapses (Kandel et al., 2013), enabled at the microlevel by the branching operation of biomolecules (section 4.2).

## 7. Biological Emergence and Physical Branching

How is it possible that goal-oriented systems and deductive logic arise out of the goal-free underlying physics? The context is the hierarchy of emergence and causation, where all the complexities of biology as outlined in section 1.2, occur. Each level of the hierarchy is equally real (Noble, 2012), and branching causation takes place at each level via complex networks of interactions which, through a combination of bottom-up and top down causation, allow organizational closure. Despite the stochasticity of what occurs, the essential core of interactions at the molecular level can be well represented as binary ON/OFF choices (Berridge, 2014). It is at the network level that these individual choices become immensely complex and able to generate the processes of life (section 1.2). How can such branching dynamics emerge from physics which by its nature does not show such branching properties (section 2)? Our main conclusion is,

*Biomolecules, and specifically proteins (Petsko and Ringe*, *2009**), provide the physical link between physics and biological causation by allowing branching dynamics at the molecular level, which can then underlie emergence of macro-scale branching dynamics and even deductive causation when incorporated in adaptive modular hierarchical networks. Both the networks and the proteins must have been shaped through processes of adaptive selection; however some of their aspects (that do not hinder their proper function) may be due to drift*.

Ion channels have been our main example, because they enable functioning of the brain, but many other biomolecules in cell signaling networks also carry out logical operations (Berridge, 2014), as do excitatory or inhibitory receptors in neurons (Kandel et al., 2013) with their synaptic thresholds. These branching functions are based in the lock and key mechanism of supra-molecular biology which enables molecular recognition (Lehn, 1995, 2007).

### 7.1. The Major Distinctions: Three Kinds of Causation

The major difference between physics and life has been characterized above as due to the difference between the immutable impersonal logic of physical causation (1) and the branching functional logic of biological causation (9), enabled by biomolecules in general and proteins in particular (section 4.2).

The progression of emergence is illustrated in Table 1. Inanimate systems are subject only to causation C1. In all life from cells to organisms to populations to ecosystems, as well as causation C1, causation C2 occurs, involving logically based branching (9) such as homeostasis (11) and adaptive selection (18). Thus causation C2 characterizes life in general (Hartwell et al., 1999) as opposed to inanimate systems. Hence there is a major difference between these two kinds of emergence out of the same basic physical elements (Ellis, 2016). What enabled causation C2 to emerge in historical terms was the origin of life out of a physical substratum, when both metabolic and adaptive evolutionary processes first came into being. We do not know how that occurred.

**Table 1:** The three major forms of causation: physical, biological, and deductive. Each relies on the previous one to enable its emergence.

	**Causation**	**Agency**	**Outcome**	**References**
C1	Physical	Physical laws	Determinist	Equation (1)
C2	Biological	Goal-seeking, Selection	Adaptive	Equations (11,18)
C3	Deductive	Logical argument	Planned outcomes	Equation (32)

However, a higher form of causation C3 occurs in intelligent life, when deductively based action (32) occurs, enabling deductive logic *per se* to have causal powers. Emergence of this kind of causation is a major transition in evolution (Maynard Smith and Szathm, 1995); we also do not know how that occurred. Intelligent organisms are those that can engage in deductive causation C3, which enables transcending the physical limitations of bodies through the power of abstract thought, prediction, planning, and imagination, enabling technology to develop (so that for example they can fly through the sky or make computer systems). It is this kind of causation (made possible by symbolic systems such as language and mathematics) that underlies the rise of civilisation and the domination of humans over the planet (Bronowski, 1973; Harford, 2017): we are no longer limited by the strength of our bodies but by the limits of our imagination and understanding.

Note that we are able to say this without having to make any specific comments on the relation between the brain and consciousness. What is indisputable is that deductive causation does indeed take place in the real world, as demonstrated by many examples (such as the existence of aircraft and computers), and is crucially different than the kind of causation characteristic of physics (section 2), although it is enabled by that kind of causation (which allows the brain to function as it does; Scott, 1995; Kandel et al., 2013).

### 7.2. Branching Physical Causation in a Biological Context

There is however a key underlying question: it is clear that branching dynamics takes place at the biomolecular level, so how then does the underlying physics allow this branching to take place? The physics *per se* does not show such branching dynamics (section 1.1); but physics in a biological context must do so, in order to allow the biological branching processes discussed here to emerge.

**Figure 6:**
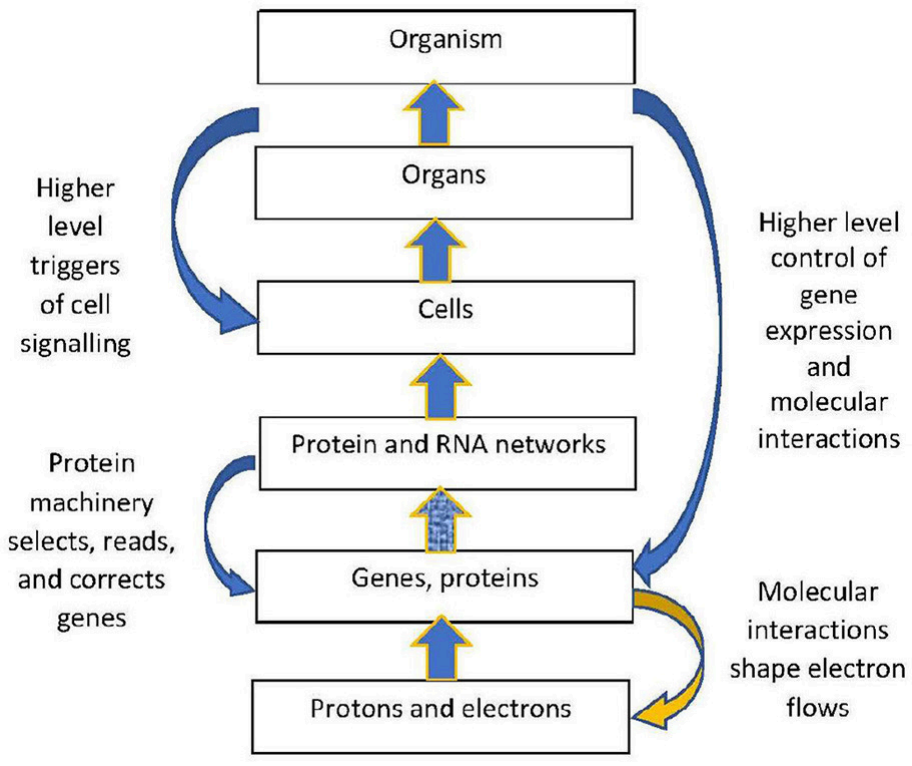
Branching physics in a biological context. Branching biological logic chains down to cause the underlying physical dynamics to branch.

The solution (Figure 6) is that top-down causation takes place (Ellis, 2016) whereby the local biomolecular context causes bifurcation of the underlying physical dynamics. Firstly, the structural constraint caused by biomolecular shape channels causation at the electron and ion level. Thus for example when a photon releases an electron in a chlorophyll molecule, that is a non-Hamiltonian process that took place because of the biological context of existence of a chlorophyll molecule in a leaf. This is underlain at the quantum level by contextual wavefunction collapse (Drossel and Ellis, 2018). Secondly, the cell signaling processes at the molecular and cellular level discussed by Berridge (Berridge, 2014) shape how electron flows take place at the underlying physical level, because when a messenger in a signaling pathway turns a process ON, that causes electrons in component molecules to flow in a structured way that would not otherwise have occurred.

In particular, such top down processes take place in the brain (Ellis, 2016; Ellis G., 2018), for example underlying the formation of memory. Eric Kandel states (Kandel, 2001), “*One of the most remarkable aspects of an animal's behavior is the ability to modify that behavior by learning*”. He then identifies how this happens at the molecular level as what he calls “*A dialogue between genes and synapses*” (Kandel, 2001). A specific event, say seeing a car crash, results in gene expression that alters synaptic strengths, which is enabled by underlying flows of electrons as indicated in Figure 6. The physics acts in such a way as to instantiate the neural connections at the neuron level needed for that memory to be stored and then available for recall at the psychological level. Neural mechanisms such as those discussed by Kandel Kandel (2001); Kandel et al. (2013) and molecular mechanisms such as discussed by Berridge Berridge (2014) enable this to happen, so what happens at the electron level is determined (up to equivalence classes) by the overall social, psychological, and mental context in a top-down way (Figure 6 omits those higher levels, but they are key parts of the causal context; Ellis, 2016).

In this way, branching physical dynamics at the bottom level emerges from the higher level branching biological dynamics (you might have seen the crash, or not; the outcomes at the electron level are affected by this contingent situation at the psychological level). Physical outcomes are determined by context, which break the symmetries of the underlying physical laws (Anderson, 1972). In the cases we consider, the relevant constraining context is the physical structure of bio-molecules in their cellular context (Hofmeyr, 2017, 2018).

**Biologically generated branching of physical outcomes:** *Biomolecules and cells shape electron flows at the physical level firstly by setting constraints on possible electron flows through their geometric shapes and dispositions (Gray and Winkler*, *2009**). Second, though signaling processes (Berridge*, *2014**) originating from higher levels (Noble*, *2012**) that shape (up to equivalence classes) what electron flows actually take place. This enables branching dynamics occurring in these signaling networks to cause branching outcomes at the electron level*.

This enables physiological processes such as those occurring in the heart (Fink and Noble, 2008) to influence electron flows at the micro-physical level through the top-down influences^14^ in physiology described by Noble (2012). Mental processes such as learning (Kandel, 2001) and deductive causation (section 6.3) can do the same, enabled by the ON/OFF operations of cell signaling networks (Berridge, 2014). The way this works during deductive argumentation (section 6.1) is similar to the way algorithms control the flow of electrons in transistors in digital computers. The branching logic of an algorithm, realized in a digital computer program, controls branching electron dynamics (which transistors are ON, allowing electron flows, or OFF, at what time) at the physical level. In that case the physical structure enabling this branching logic at the electron level is the junctions between different layers in transistors^15^.

**Biology-physics closure of constraints**. *Extension of the needed functional closure of constraints in biology (section 1.2, Mossio and Moreno*, *2010**; Montévil and Mossio*, *2015**) to the underlying physics level is provided by the fact that the branching biological logic at higher levels, including cellular (Rhoades and Pflanzer*, *1989**; Randall et al.*, *2002**; Berridge*, *2014**), and mental (Kandel*, *2001**) functioning, induces congruent branching dynamics at the underlying physical level by changing constraints at that level*.

Equation (2) has to be replaced by

(35) $$\begin{array}{r} C\left(c\left(t\right),\text{X },t\right)=C\left(t\right) \end{array}$$

where the time-dependent nature of the physics constraints derives from the time-dependent biological context, and means that the physics evolution is no longer subject to the uniqueness theorems mentioned in sections 2.1, 3.1. This has to be so in order that the biology-physics relation be consistent.

A physics analogy is a pendulum made of a bob of mass *m* that is constrained to move on a circular arc by a string of length *L*(*t*) that varies with time (this is the constraint *C*(*t*) governing possible motions of the bob) (Feldman, 2007) see the Appendix. The evolution is determined by the macroscopic constraint *C*(*t*), which controls outcomes at both macro and micro levels in a way that cannot be predicted from a knowledge of the initial data (starting position **X**_0_ and speed **v**_0_) alone. The dynamics can be controlled by an experimental protocol for *L*(*t*) designed by a scientist (which is top down causation DEOC from the mental level as in section 6.3), or can be unpredictable even in principle, when *L*(*t*) is controlled by a computer receiving signals from a detector of particles emitted by decay of a radioactive element (cf. section 2.3).

## Author Contributions

GE provided the main idea and drafted the main text. JK contributed further ideas and helped develop the text.

### Conflict of Interest Statement

The authors declare that the research was conducted in the absence of any commercial or financial relationships that could be construed as a potential conflict of interest.

## Footnotes

^2^We do not attempt to deal in this article with the vexed issue of how life started in the first place. Thus we do not discuss how compartmentalization, metabolism, or adaptive selection and associated genetic information came into being. We assume that they are already in place, and propose that our discussion is then a valid representation of the difference between physics and biology in that context.

^3^It will in some cases be appropriate to call this top-down causation. That usage is controversial: it will be defended in a forthcoming paper (Ellis and Gabriel, 2019).

^4^Although this is effectively true in some specific contexts in developmental biology, where pre-determined developmental stages occur at specific times in an organisms developmental history Gilbert (2006); Wolpert (2002) via specific mechanisms whereby “*groups of cells are progressively apportioned distinct fates through a process of cell specification*” (Berridge, 2014: Module 8). For example, gastrulation occurs at a specific stage of development (Wolpert, 2002).

^5^There may also be intermediate states of channel opening. Then the logic is (10) rather than (9).

^6^The concept of information is contentious in biology (Godfrey-Smith and Sterelny, 2016; Koonin, 2016). However, signaling is not (Berridge, 2014). We will take the pragmatic view that signals convey information.

^7^See for example “*Regulation of Ca*^2+^ *homoeostasis by multiple hormonal and organ effector systems*” in Berridge (2014): Module 7, p. 76, and “*Hormonal regulation of blood Na*^+^ *levels*” in Berridge (2014): Module 7 , p. 105.

^8^In practice, the response function is not discontinuous as in this representation, but is a a logistic curve linking ‘ON’ and ‘OFF’ states. The principle remains the same: but one now uses a more complex response function. Equation (14) is a good first approximation (cf. Berridge, 2014.)

^9^We note here that positive sexual selection may also have played a role

^10^Or perhaps at the group level; this is the contentious issue of levels of selection (Okasha, 2010)

^11^We note here that these processes can become automated after much practice so that they are intuitive rather than the result of directed mental effort. Nevertheless the nature of the causation is the same.

^12^We are not giving a formal definition of logic here, but rather a sketch of how it works. It can be any form of logic that has been discovered by the human mind.

^13^Strictly speaking, the word “necessarily” is superfluous. We add it for emphasis here and below. A similar remark applies to “probably” in (26).

^14^Philosophical objections to this possibility based in the idea of supervenience are countered in (Ellis, 2016) and will be fully refuted in a forthcoming paper (Ellis and Gabriel, 2019).

^15^See for example *Bipolar junction transistor* in Wikipedia.

## Appendix

### Pendulum With Varying Length

The dynamic equations for a pendulum of varying length are set out clearly in Feldman (Feldman, 2007). A bob on an idealised massless rod swings back and forth about a hinge; the rod angle from the vertical at the hinge is θ(*t*). The bob mass is *M*. The bob can slide up or down the rod, so the length *L*(*t*) from the hinge to the bob in general varies: $\overset{\cdot }{L}\left(t\right)\neq 0$ for some time *t*. The bob position (*x*(*t*), *y*(*t*)) at time *t* relative to the hinge is

(A1) $$\begin{array}{r} x\left(t\right)=L\left(t\right)\sin\theta \left(t\right),y\left(t\right)=-L\left(t\right)\cos\theta \left(t\right), \end{array}$$

giving the constraint equation (cf. Eqn.(35))

(A2) $$\begin{array}{r} L\left(t\right)=\sqrt{x{\left(t\right)}^{2}+y{\left(t\right)}^{2}}. \end{array}$$

The kinetic energy *T*(*t*) and potential energy *U*(*t*) are

(A3) $$\begin{array}{r} T\left(t\right)=\frac{1}{2}M\left[\overset{\cdot }{L}{\left(t\right)}^{2}+L{\left(t\right)}^{2}\overset{\cdot }{\theta }{\left(t\right)}^{2}\right],V\left(t\right)=-MgL\left(t\right)\cos\theta \left(t\right) \end{array}$$

The Lagrangian is

(A4) $$\begin{array}{r} L\left(t\right)=T\left(t\right)-U\left(t\right)=M\left[\frac{1}{2}\left\{\overset{\cdot }{L}{\left(t\right)}^{2}+L{\left(t\right)}^{2}{\overset{\cdot }{\theta }}^{2}\left(t\right)\right\}+gL\left(t\right)\cos\theta \left(t\right)\right] \end{array}$$

and the Lagrange equation of motion

(A5) $$\begin{array}{r} \frac{d}{dt}\left(\frac{\partial L}{\partial \overset{\cdot }{\theta }}\right)-\frac{\partial L}{\partial \theta }=0 \end{array}$$

shows that

(A6) $$\begin{array}{r} \frac{d^{2}\theta }{dt^{2}}\left(t\right)+2\frac{\overset{\cdot }{L}\left(t\right)}{L\left(t\right)}\overset{\cdot }{\theta }\left(t\right)+\frac{g}{L\left(t\right)}\sin\theta \left(t\right)=0, \end{array}$$

reducing to the standard pendulum equation when $L\left(t\right)=L_{0}\Leftrightarrow \overset{\cdot }{L}\left(t\right)=0$. The initial data $\left(\theta \left(t_{0}\right),\overset{\cdot }{\theta }\left(t_{0}\right)\right)$ does not determine the solution θ(*t*) for *t* > *t*_1_ if $\overset{\cdot }{L}\left(t\right)\neq 0$ at any time *t*_1_ > *t*_0_, because of this time-variation of the constraint *L*(*t*).
